# Serum cytokine and chemokine profiles and disease prognosis in hepatitis B virus-related acute-on-chronic liver failure

**DOI:** 10.3389/fimmu.2023.1133656

**Published:** 2023-04-27

**Authors:** Bingbing Zhu, Fangyuan Gao, Yuxin Li, Ke Shi, Yixin Hou, Jialiang Chen, Qun Zhang, Xianbo Wang

**Affiliations:** Center of Integrative Medicine, Beijing Ditan Hospital, Capital Medical University, Beijing, China

**Keywords:** prospective cohort study, Luminex, prognostic model, CXCl2, IL-8, IL-6, 90-day prognosis, HBV-ACLF

## Abstract

**Background:**

Hepatitis B virus-related acute-on-chronic liver failure (HBV-ACLF) has significant morbidity and mortality and is associated with the induction of cytokines/chemokines, which might contribute to the pathogenesis of liver injury. This study aimed to explore the cytokine/chemokine profiles of patients with HBV-ACLF and develop a composite clinical prognostic model.

**Methods:**

We prospectively collected blood samples and the clinical data of 107 patients with HBV-ACLF admitted to the Beijing Ditan Hospital. The concentrations of 40-plex cytokines/chemokines were measured in 86 survivors and 21 non-survivors using the Luminex assay. Discrimination between the cytokine/chemokine profiles in different prognosis groups was analyzed using the multivariate statistical techniques of principal component analysis (PCA) and partial least squares discriminant analysis (PLS-DA). An immune-clinical prognostic model was obtained using multivariate logistic regression analysis.

**Results:**

The PCA and PLS-DA indicated that cytokine/chemokine profiling could clearly distinguish patients with different prognoses. A total of 14 cytokines, namely, IL-1β, IL-6, IL-8, IL-10, TNF-α, IFN-γ, CXCL1, CXCL2, CXCL9, CXCL13, CX3CL1, GM-SCF, CCL21, and CCL23, were significantly correlated with disease prognosis. Multivariate analysis identified CXCL2, IL-8, total bilirubin, and age as independent risk factors that constituted the immune-clinical prognostic model, which showed the strongest predictive value of 0.938 compared with those of the Chronic Liver Failure Consortium (CLIF-C) ACLF (0.785), Model for End-Stage Liver Disease (MELD) (0.669), and MELD-Na (0.723) scores (*p* < 0.05 for all).

**Conclusion:**

The serum cytokine/chemokine profiles correlated with the 90-day prognosis of patients with HBV-ACLF. The proposed composite immune-clinical prognostic model resulted in more accurate prognostic estimates than those of the CLIF-C ACLF, MELD, and MELD-Na scores.

## 1 Introduction

Acute-on-chronic liver failure (ACLF) is a complex syndrome characterized by the acute deterioration of liver function in patients with known chronic liver disease, which is characterized by acute onset, rapid progression, and dramatically high short-term mortality. The latest data suggest that approximately 250 million people are infected with hepatitis B virus (HBV) worldwide (1), 75% of whom are in Asia (2), with HBV-related ACLF (HBV-ACLF) accounting for over 70% of the ACLF cases in the Asia-Pacific region (3). With the popularization of hepatitis B vaccination and the application of antiviral drugs, the incidence of HBV-ACLF has decreased year by year. However, owing to the lack of effective drugs to block the acute and large-scale necrosis of hepatocytes, the mortality rate of HBV-ACLF remains high. Consequently, reliable biomarkers are needed to estimate the disease prognosis and patient survival in order to guide the curative management of HBV-ACLF.

Several studies have reported systemic inflammation in patients with HBV-ACLF, in which immune imbalance is an important part of the pathogenesis and disease progression of ACLF and is manifested by excessive innate immune activation, leading to a cytokine storm (4). The excessive inflammatory state is caused by the activation of the cells of the innate immune system in response to pathogen- and damage-associated molecular patterns (PAMPs and DAMPs, respectively). This causes the release of a variety of effector molecules, such as inflammatory cytokines and chemokines, among others, which leads to a heightened inflammatory state in the body (5). The complicated interaction networks of many inflammatory molecules with negative and feedback mechanisms regulate the pathological process of ACLF, and their fine calibration determines the outcomes of patients (6, 7). In another study by us (not yet published), it was found that, compared to asymptomatic HBV carriers and chronic hepatitis B patients, a number of soluble immune factors were elevated in patients with HBV-ACLF. The multiple abnormally expressed soluble immune components reflected a broad and complicated immune dysfunction in HBV-ACLF. Although a number of inflammatory factors in patients with ACLF have been reported in a few studies, a comprehensive study of the cytokine/chemokine profiles is nonetheless yet to be undertaken.

Clinical data including the levels of alanine aminotransferase (ALT), aspartate aminotransferase (AST), and total bilirubin (TBIL) and its international normalized ratio (INR), as well as multivariable prognostic models based on clinical data obtained from the Chronic Liver Failure Consortium (CLIF-C) ACLF (8), the Chinese Group on the Study of Severe Hepatitis B-ACLF (COSSH-ACLF) criteria (9), the model for end-stage liver disease (MELD) (10), and MELD-Na (11), are useful for the assessment of disease severity and prognosis to guide treatment; however, there are some limitations. The Luminex assay, which is based on a high-throughput cytokine bead assay, provides a powerful tool for identifying the key markers of inflammation associated with disease severity. This technology allows researchers to measure the cytokine concentrations in a variety of biological samples quickly and accurately, enabling them to better understand the relationship between inflammation and disease progression and to offer rational intervention strategies. This study sought to assess 40 host inflammatory molecules using the Luminex assay and analyze their correlation with the disease outcomes, with the purpose of finding biomarkers and establishing a composite immune-clinical prognostic model.

## 2 Materials and methods

### 2.1 Study and patient characteristics

This is a prospective observational study. From January 2018 to January 2022, a total of 107 HBV-ACLF inpatients from Beijing Ditan Hospital, Capital Medical University, were recruited. Blood samples were collected for cytokine determination. Patients who were <18 or >80 years of age; those with a history of liver malignancies or other tumors; with decompensated liver cirrhosis; those who had viral hepatitis or other viral infections, including human immunodeficiency virus, cytomegalovirus, and Epstein–Barr virus co-infection; with autoimmune liver disease, liver transplantation, and severe chronic extrahepatic disease; and those who were pregnant were excluded from the study. Ethical approval for this study was obtained from the Ethics Committee of Beijing Ditan Hospital (Beijing, China), and all participants provided written informed consent.

### 2.2 Definitions

The criteria of the Asian Pacific Association for the Study of the Liver (APASL) for ACLF were as follows: acute hepatic insult manifesting as jaundice, with TBIL ≥5 mg/dl (85 μmol/L), and coagulopathy, with INR ≥1.5 or prothrombin activity (PTA) <40%, complicated within 4 weeks by clinical ascites and/or hepatic encephalopathy (HE) in a patient with previously diagnosed or undiagnosed chronic liver disease/cirrhosis (12). Prognostic models, including the CLIF-C ACLF (8), COSSH-ACLF II (9), MELD (10), and MELD-Na scores (11), were calculated according to previously published criteria. All models and definitions were applied at the time of the enrollment of patients in this study.

### 2.3 Treatment schedules

All of the patients included in this study received standard medical treatment to eliminate or control the precipitating factors and associated complications during hospitalization, which included bed rest, nutritional support, and antiviral therapy, among others. The specific treatment strategies are described in the guidelines (13). All patients were followed from the time of their diagnosis until either their death or the end of the 90-day follow-up period.

### 2.4 Data and sample collection

We collected the following clinical and demographic information of the participants: age, gender, incidence of complications [e.g., ascites, spontaneous bacterial peritonitis (SBP), and HE], and laboratory indicators [i.e., ALT, AST, TBIL, serum albumin (ALB), gamma-glutamyl transpeptidase (GGT), cholinesterase (CHE), serum sodium (Na), serum potassium (K), serum creatinine (Cr), white blood cell (WBC), neutrophil count (NC), lymphocyte count (LC), neutrophil-to-lymphocyte ratio (NLR), monocyte count (MC), monocyte-to-lymphocyte ratio (MLR), red blood cell (RBC), platelet (PLT) count, prothrombin time (PT), PTA, and (INR)]. Blood samples were obtained on admission and centrifuged at 3,500 rpm for 10 min at 4°C. The serum samples thus obtained were stored at −80°C.

### 2.5 Analyses of 40-plex cytokines/chemokines using the Luminex system

The expression levels of 40 cytokines/chemokines (i.e., IL-1β, IL-2, IL-4, IL-6, IL-8, IL-10, IL-16, TNF-α, IFN-γ, GM-CSF, CCL1/I-309, CCL2/MCP-1, CCL3, CCL7, CCL8, CCL11, CCL13, CCL15, CCL17, CCL19, CCL20, CCL21, CCL22, CCL23, CCL24, CCL25, CCL26, CCL27, CXCL1, CXCL2, CX3CL1, CXCL5, CXCL6, CXCL9, CXCL10, CXCL11, CXCL12, CXCL13, CXCL16, and MIF) were measured using the Bio-Plex Pro Human Chemokine Panel 40-plex kit (#1171AK99MR2) with the Bio-Plex 200 system (Bio-Rad, Hercules, CA, USA) by Wayen Biotechnologies (Shanghai, China). In brief, the serum samples were centrifuged at 1,000 rpm for 10 min and the supernatants collected and diluted fourfold. Subsequently, diluted samples (50 μl) were incubated in 96-well plates with microbeads for 1 h and then incubated with the detection antibody for 30 min. Furthermore, streptavidin-phycoerythrin (PE) was added in each well and incubated for 10 min. The concentrations of the cytokines in the samples were analyzed using the standards that were run in each of the plates. Finally, the data acquired were analyzed using the Bio-Plex Manager 6.0 software. Each run contained appropriate quality controls run in duplicate.

### 2.6 Principal component analysis and partial least squares discriminant analysis

The 40-plex cytokine/chemokine bead array data were analyzed using the MetaboAnalyst 5.0 software (available online at http://www.metaboanalyst.ca) (14) for the multivariate principal component analysis (PCA) and partial least squares discriminant analysis (PLS-DA) based on R. PCA was utilized to investigate the general cluster. Subsequently, the supervised orthogonal PLS-DA was employed to further distinguish between different groups.

### 2.7 Statistical analysis

Concentrations above or below the detection limit were assigned as the highest or lowest values, respectively, from the respective standard curves. For statistical analysis, concentrations below the detection limit were converted to a value of 0.5×, the lowest value of the standard curve. Categorical variables were presented as percentages and were compared using the chi-square test or Fisher’s exact test. Continuous variables were shown as the mean ± standard deviation or median (interquartile range) and were compared using Student’s *t*-test or the Mann–Whitney *U* test. Pearson’s correlation analysis was performed to assess the correlation between the cytokine/chemokine levels and clinical parameters. Logistic regression analysis was used for multivariate analysis. Univariate analysis was screened at *p* < 0.05 as the test level from baseline data, and logistic multifactor regression analysis was performed with the backward step likelihood ratio method (step probability *α* = 0.05). The area under the receiver operating characteristic curve (AUROC) was calculated to evaluate the predictive value of the different variables and models. The AUROCs between models were compared using the DeLong test (15). Statistical analyses were performed using SPSS software (version 24.0; Chicago, IL, USA). Heatmaps were generated using R software (version 4.2.1; https://www.r-project.org). *P* < 0.05 was considered as statistically significant.

## 3 Results

### 3.1 Clinical characteristics of HBV-ACLF

In total, 107 patients with HBV-ACLF were recruited for analysis. Of these patients, 21 (19.6%) died within 90 days, while 86 (80.4%) survived. The baseline clinical characteristics of the study population are shown in **Table 1**. Univariate analysis showed that age, HE, SBP, TBIL, Na, Cr, WBC, NC, NLR, MC, MLR, PT, PTA, INR, and the CLIF-C ACLF, COSSH-ACLF II, MELD, and MELD-Na scores were significantly associated with short-term mortality (*p* < 0.05).

**Table 1:** Analysis of the clinical characteristics of the two groups.

	Total (*n* = 107)	>Survivors (*n* = 86)	Non-survivors (*n* = 21)	*p*
Age (years)	43.59 ± 11.8	41.90 ± 11.56	50.10 ± 10.61	0.005^a^
Gender	88/19	68/18	20/1	0.082
HE (%)	45 (42.10)	31 (44.90)	14 (66.70)	0.011^c^
Acites (%)	79 (73.80)	61 (80.30)	18 (85.70)	0.167
SBP (%)	44 (41.10)	28 (32.60)	16 (76.20)	0.012^c^
ALT (U/L)	380.80 (117.70–775.40)	322.10 (101.25–750.65)	412.85 (122.60–895.28)	0.509
AST (U/L)	217.30 (117.85–514.25)	199.10 (103.45–479.05)	308.40 (204.43–691.90)	0.051
TBIL (μmol/L)	283.82 ± 137.02	261.83 ± 122.09	368.46 ± 160.41	0.002^a^
ALB (g/L)	31.86 ± 4.03	31.80 ± 4.05	32.10 ± 4.06	0.768
GGT (U/L)	88.80 (56.90–148.93)	88.80 (54.38–148.93)	84.45 (57.88–170.08)	0.683
CHE (U/L)	3,478.12 ± 1,436.09	3,411.04 ± 1,434.00	3,725.26 ± 455.33	0.401
Na (mmol/L)	137.69 ± 4.40	138.46 ± 3.59	134.71 ± 5.88	0.012^a^
K (mmol/L)	3.79 ± 0.53	3.74 ± 0.52	3.96 ± 0.52	0.108
Cr (μmol/L)	70.16 ± 37.48	69.21 ± 20.48	102.93 ± 66.85	0.037^a^
WBC (10^9^/L)	4.90 (3.82–7.49)	5.48 ± 2.53	7.91 ± 4.12	0.020^a^
NC (10^9^/L)	2.99 (2.24–5.01)	2.87 (1.99–4.75)	4.03 (2.97–8.75)	0.005^b^
LC (10^9^/L)	1.32 ± 0.76	1.36 ± 0.75	1.13 ± 0.77	0.216
NLR	2.54 (1.91–4.43)	2.40 (1.72–3.67)	4.77 (2.42–10.22)	<0.001^b^
MC (10^9^/L)	0.63 ± 0.39	0.57 ± 0.32	0.87 ± 0.52	0.002^a^
MLR	0.46 (0.30–0.66)	0.40 (0.27–0.59)	0.79 (0.56–1.36)	<0.001^b^
RBC (10^12^/L)	3.88 ± 0.85	3.85 ± 0.84	4.027 ± 0.89	0.436
PLT (10^9^/L)	106.49 ± 51.27	102.38 ± 47.56	122.30 ± 62.51	0.123
PT (s)	24.00 (20.85–28.20)	23.60 (20.75–27.10)	26.15 (22.15–37.45)	0.042^b^
PTA (%)	36.00 ± 10.54	37.16 ± 9.78	31.55 ± 12.38	0.033^a^
INR	2.33 ± 0.71	2.21 ± 0.52	2.81 ± 1.09	0.027^a^
COSSH-ACLF II score	6.54 ± 1.09	6.26 ± 0.80	7.67 ± 1.38	<0.001^a^
CLIF-C ACLF score	36.99 ± 6.28	35.60 ± 5.46	41.99 ± 6.63	<0.001^a^
MELD	22.86 ± 4.96	21.90 ± 3.53	26.41 ± 7.47	0.019^a^
MELD-Na	24.05 ± 6.92	22.29 ± 4.20	30.51 ± 049	0.003^a^

HE, hepatic encephalopathy; SBP, spontaneous bacterial peritonitis; ALT, alanine aminotransferase; AST, aspartate aminotransferase; TBIL, total bilirubin; ALB, serum albumin; GGT, gamma-glutamyl transpeptidase; CHE, cholinesterase; Cr, blood creatinine level; WBC, white blood cell; NC, neutrophil count; LC, lymphocyte count; NLR, neutrophil/lymphocyte ratio; MC, monocyte count; MLR, monocyte/lymphocyte ratio; RBC, red blood cell; PLT, platelets; PT, prothrombin time; PTA, prothrombin activity; INR, international normalized ratio; COSSH, Study of Severe Hepatitis B; CLIF-C OF, Chronic Liver Failure Consortium organ failure; CLIF-C ACLF, Chronic Liver Failure Consortium acute-on-chronic liver failure; MELD, Model for End-Stage Liver Disease; MELD-Na, Model for End-Stage Liver Disease and sodium.

*p < 0.05 (survivors vs. non-survivors);

a P-values comparing the survivor group and the mortality group from the t-test;

b Mann–Whitney U test;

c Chi-square test.

### 3.2 Cytokine/chemokine profiles of HBV-ACLF

**Figure 1:**
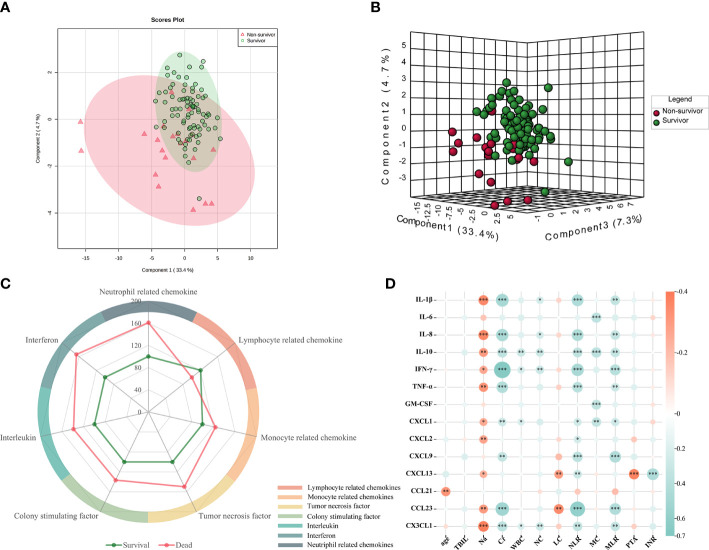
Comparison of the serum cytokines between non-survivors and survivors. **(A, B)** Principal component analysis (PCA) and partial least squares discriminant analysis (PLS-DA) of the cytokine expression profiles in survivors and non-survivors. The *red circle* represents the multiplex cytokine expression for non-survivors, while the *green color* depicts survivors. **(C)** Radar map of the six classification scores in non-survivors (*n* = 21) compared with those in survivors (*n* = 86). The *radius* represents the percentage of expression. Interleukins: IL-1β, IL-2, IL-4, IL-6, IL-10, and IL-16; monocyte-associated chemokines: CCL2, CCL3, CCL7, CCL8, CCL13, and CCL15; neutrophil-related chemokines: CXCL1, CXCL2, CXCL5, CXCL6, CXCL8, and CXCL10; and lymphocyte-related chemokines: CCL1, CCL2, CCL17, CCL21, CCL22, and CCL23. **(D)** Correlation heatmap of the significantly different 12 clinical indices and 14 differentially expressed serum cytokines/chemokines. *Red*: positive correlation; *blue*: negative correlation. *Asterisk* denotes *p*-values that show significant correlation.

The levels of 40 serum cytokines/chemokines were measured using the Luminex assay, and analysis of the cytokine/chemokine profiles of the survivors (*n* = 86) and non-survivors (*n* = 21) was conducted. The PCA methodology is considered as an unbiased statistical method of research. We therefore utilized this method to analyze the tendency of the cytokine/chemokine profiles in the different groups. As **Figure 1** shows, there was a clear distinction between the survivors and non-survivors from the PCA (**Figure 1A**). On the other hand, the PLS-DA score plots could be thoroughly separated in the different prognosis groups (**Figure 1B**). Furthermore, to depict the overall differences, the classification scores of the six categories were determined by computing the weighted mean expression levels of the immune factors in each classification. A radar map revealed that the patients who died displayed significantly higher levels of interleukins, tumor necrosis factor, interferon, colony-stimulating factor, and monocyte-associated and neutrophil-related chemokines. However, the serum levels of lymphocyte chemokines were markedly decreased in non-survivors (**Figure 1C**). These data strongly implied that it is possible to search for potential biomarkers to distinguish patients with different prognoses.

### 3.3 Cytokine/chemokine biomarker screening and the correlations with clinical parameters

**Figure 2:**
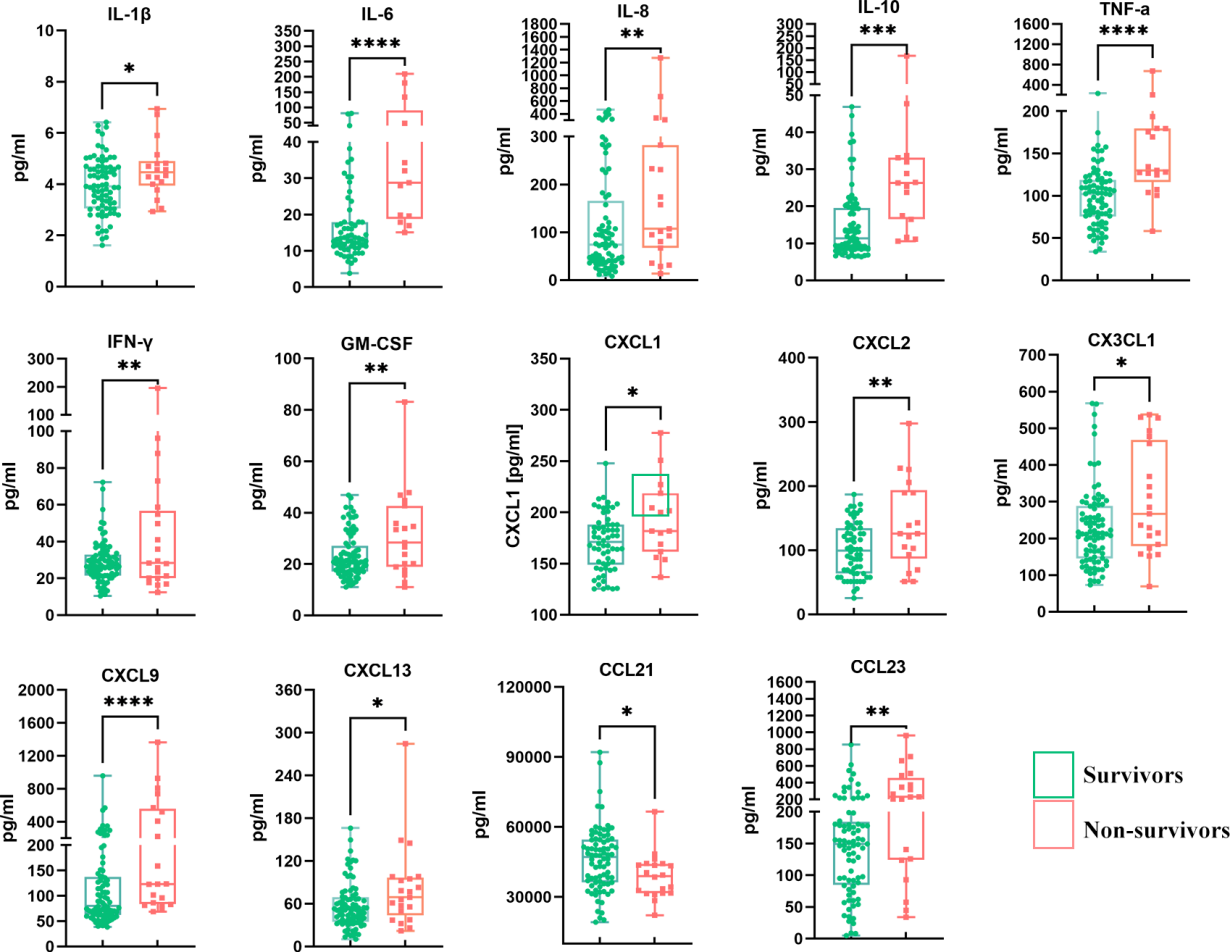
Comparison of the 14 differentially expressed serum cytokines/chemokines between survivors and non-survivors. *p < 0.05, **p < 0.01, ***p < 0.001, ****p < 0.0001.

In the univariate analysis, 14 of the 40 detected cytokines/chemokines, namely, IL-1β, IL-6, IL-8, IL-10, TNF-α, IFN-γ, GM-CSF, CXCL1, CXCL2, CX3CL1, CXCL9, CXCL13, CCL21, and CCL23, were found to be significantly associated with 90-day prognosis (*p* < 0.05) (**Table 2**, **Figure 2**). To further determine the relationship between serum cytokines/chemokines and the clinical parameters in HBV-ACLF, a correlation heatmap was constructed. As shown in **Figure 1D**, Na was negatively correlated with most of the 14 cytokines/chemokines. On the other hand, Cr was positively correlated with increased serum levels of IL-1β, IL-8, IL-10, TNF-α, IFN-γ, CXCL1, CXCL9, CX3CL1, and CCL23. Our previous study showed that NLR can significantly predict the prognosis of patients with HBV-ACLF (16). NLR was negatively correlated with increased serum levels of IL-1β, IL-8, IL-10, TNF-α, IFN-γ, CXCL1, CXCL9, CXCL13, CX3CL1, and CCL23. MLR was positively correlated with increased serum levels of IL-1β, IL-8, IL-10, TNF-α, IFN-γ, CXCL1, CXCL9, CX3CL1, and CCL23. PTA was negatively correlated with CXCL13, while INR was positively correlated with CXCL13 (all *p* < 0.05).

**Table 2:** Analysis of the inflammatory cytokine and chemokine profiles of the two groups.

Variable (pg/ml)	Total (*n* = 107)	Survivors (*n* = 86)	Non-survivors (*n* = 21)	*p*
IL-1β	4.00 (3.31–4.73)	3.91 (3.15–4.68)	4.57 (3.93–5.23)	0.009*
IL-2	7.39 ± 3.86	7.11 ± 2.39	8.56 ± 7.26	0.378
IL-4	81.76 ± 28.35	80.02 ± 27.91	88.92 ± 29.69	0.198
IL-6	12.65 (7.52–19.61)	11.99 (7.41–17.15)	19.61 (7.99–41.13)	0.015*
IL-8	98.38 (43.98–226.79)	82.28 (40.47–174.52)	224.31 (118.92–330.82)	<0.001*
IL-10	13.12 (8.62–22.40)	10.95 (8.38–19.66)	26.30 (13.31–33.38)	<0.001*
IL-16	131.21 (83.50–233.36)	128.11 (81.86–230.46)	153.83 (110.96–371.30)	0.076
TNF-α	108.51 ± 65.04	98.89 ± 34.09	147.36 ± 124.32	0.002*
IFN-γ	27.81 (21.696–35.83)	26.46 (21.46–32.86)	36.25 (24.56–56.74)	0.008*
GM-CSF	21.35 (17.67–33.42)	20.75 (17.51–28.85)	31.34 (19.61–43.68)	0.007*
CCL1/I-309	79.02 ± 29.55	75.96 ± 22.72	91.55 ± 47.21	0.155
CCL2/MCP-1	117.79 (92.80–136.53)	120.82 (96.31–135.86)	98.59 (86.65–145.77)	0.265
CCL3/MIP-1α	15.22 (10.41–29.78)	14.29 (10.22–29.15)	20.89 (12.44–47.01)	0.152
CCL7/MCP-3	129.76 (95.59–233.48)	129.76 (95.59–196.04)	175.17 (86.08–320.42)	0.207
CCL8/MCP-2	22.51 (9.99–43.02)	23.78 (8.90–46.47)	18.60 (10.36–39.58)	0.505
CCL11/Eotaxin	87.11 ± 25.51	86.71 ± 22.74	88.77 ± 35.32	0.742
CCL13/MCP-4	53.50 (37.60–71.10)	52.52 (37.50–67.48)	69.98 (36.74–76.41)	0.293
CCL15/MIP-1d	2,071.81 ± 903.93	2,013.49 ± 800.52	2,310.49 ± 1,237.95	0.305
CCL17/TARC	9.50 (3.77–43.42)	12.66 (3.77–45.79)	4.66 (3.77–28.16)	0.364
CCL19/MIP-3b	394.42 ± 181.69	366.09 ± 47.05	402.61 ± 18.19	0.707
CCL20/MIP-3a	35.86 (22.08–64.17)	32.63 (19.94–61.70)	47.95 (31.43–67.83)	0.069
CCL21 (×10^3^)/6Ckine	44.94 (35.53–54.93)	48.01 (36.53–56.65)	39.37 (31.65–44.31)	0.015−
CCL22/MDC	253.91 ± 131.84	265.00 ± 129.95	208.48 ± 132.82	0.078
CCL23/MPIF-1	153.31 (83.66–215.33)	149.05 (83.47–180.48)	229.24 (108.17–429.47)	0.023*
CCL24/Eotaxin-2	202.11 (79.02–339.88)	240.95 ± 39.02	256.45 ± 202.22	0.621
CCL25/TECK	1,196.92 (934.10–1,453.90)	1,139.93 (931.82–1,389.74)	1,369.58 (879.56–1,919.75)	0.204
CCL26/Eotaxin-3	116.28 (90.64–165.25)	113.31 (92.16–160.28)	136.15 (72.07–202.95)	0.470
CCL27/CTACK	764.12 ± 385.93	738.56 ± 394.21	868.82 ± 338.54	0.167
CXCL1/Gro-α	167.51 (145.57–189.93)	164.56 (144.37–185.26)	181.74 (154.93–2,222.85)	0.025*
CXCL2/Gro-β	105.14 (84.60–141.02)	102.28 (73.81–128.38)	142.80 (113.00–189.66)	<0.001*
CX3CL1/Fractalkine	243.98 ± 119.98	229.39 ± 108.37	303.77 ± 147.36	0.039*
CXCL5/ENA-78	2,415.66 (1,758.41–3,993.61)	2,338.449 (1,692.73–3,764.05)	3,865.65 (1,983.48–6,016.35)	0.089
CXCL6/GCP-2	97.65 (69.00–152.29)	95.38 (69.00–159.29)	112.87 (71.02–156.77)	0.605
CXCL9/MIG	88.95 (66.58–151.36)	80.41 (61.97–134.52)	123.20 (82.78–546.95)	0.006*
CXCL10/IP-10	428.08 (221.21–815.41)	461.70 (243.34–819.73)	374.61 (191.47–781.37)	0.515
CXCL11/ITAC	144.69 (89.44–255.77)	152.46 (93.12–237.54)	123.12 (80.59–399.01)	0.817
CXCL12/SDF1	2138.53 ± 609.37	2101.22 ± 582.77	2291.32 ± 702.95	0.201
CXCL13/BCA-1	53.29 (37.06–76.92)	51.48 (34.52–69.01)	69.07 (43.68–96.39)	0.036*
CXCL16/SCYB16	349.92 ± 159.09	335.63 ± 141.39	408.47 ± 211.44	0.060
MIF	2,191.70 (1,434.44–3,444.30)	2,184.56 (1,451.15–3,356.15)	2,267.01 (1,327.78–5,106.52)	0.663

IL, interleukin; TNF-α, tumor necrosis factor alpha; IFN-γ, interferon gamma; GM-CSF, granulocyte-macrophage colony stimulating factor; CCL, chemokine (C–C motif) ligand; MCP-1, monocyte chemotactic protein-1; MIP-1α, macrophage inflammatory protein-1α; MCP-2, monocyte chemotactic protein-2; MCP-3, monocyte chemotactic protein-3; MIP-1β, macrophage inflammatory protein-1β; MDC, macrophage-derived chemokines; MCP-4, monocyte chemotactic protein-4; MPIF-1, myeloid inhibitory factor 1; TECK, thymus-expressed chemokines; CTACK, cutaneous T-cell-attracting chemokine; CXCL, chemokine (C–X–C motif) ligand; GRO-α, growth-regulated oncogene-α; GRO-β, growth-regulated oncogene-β; ENA-78, epithelial neutrophil-activating peptide-78; GCP-2, granulocyte chemotactic protein-2; MIG, monokine induced by IFN-γ; IP-10, interferon-inducible protein-10; ITAC, IFN-inducible T-cell alpha chemoattractant; SDF1, stromal-derived factor 1; BCA-1, B-cell-attracting chemokine 1; MIF, migration inhibitor factor.

*p < 0.05, survivors vs. non-survivors.

### 3.4 Derivation of prognostic models of HBV-ACLF

The variables that were different between patients with varied outcomes were evaluated to identify independent predictors of disease progression. The univariate analysis showed that nine clinical indicators (i.e., age, HE, SBP, TBIL, Na, Cr, NLR, MLR, and PTA) and 14 cytokines/chemokines (i.e., IL-1β, IL-6, IL-8, IL-10, TNF-α, IFN-γ, GM-CSF, CXCL1, CXCL2, CX3CL1, CXCL9, CXCL13, CCL21, and CCL23) were significantly associated with 90-day prognosis (**Tables 1**, **2**). The aforementioned variables were then entered into multivariate regression analyses, with only three clinical indicators and three cytokines/chemokines remaining independent risk factors for the mortality of patients with HBV-ACLF. The results of the multivariate logistic regression analysis are shown in **Table 3** and **Supplementary Tables S1**, **S2**. As shown in **Table 3**, age (OR = 1.092, 95% CI = 1.021–1.167, *p* = 0.010), TBIL (OR = 1.005, 95% CI = 1.000–1.010, *p* = 0.045), and NLR (OR = 1.579, 95% CI = 1.163–2.145, *p* = 0.003) were found to be independently related to 90-day mortality in HBV-ACLF. On the basis of the regression coefficients of these three independent variables associated with hospital mortality according to the multivariate logistic analysis, a clinical prognostic model for patients with HBV ACLF was derived using the following mathematical formula: Ditan clinical model = −8.967 + 0.088 × Age + 0.005 × TBIL (μmol/L) + 0.457 × NLR.

**Table 3:** Multivariate logistic analyses of the cytokines/chemokines and the clinical factors.

Factor		*β*	OR (95% CI)	*p*	Model
Clinical factors	Age	0.088	1.092 (1.021–1.167)	0.010	Clinical model = −8.967 + 0.088 × Age + 0.005 × TBIL (μmol/L) + 0.457 × NLR
	TBIL	0.005	1.005 (1.000–1.010)	0.045	
	NLR	0.457	1.579 (1.163–2.145)	0.003	
Cytokines	CXCL2	0.024	1.024 (1.009–1.039)	0.001	Immune model = −5.379 + 0.024 × CXCL2 + 0.023 × IL-6 + 0.014 × IL-8
	IL-6	0.023	1.023 (1.000–1.047)	0.054	
	IL-8	0.014	1.014 (1.006–1.022)	< 0.001	
Clinical factors and cytokines	Age	0.139	1.149 (1.036–1.274)	0.008	Immune-clinical model = −21.820 + 0.139 × Age + 0.013 × TBIL (μmol/L) + 0.061 × CXCL2 + 0.015 × IL-8
	TBIL	0.013	1.014 (1.004–1.023)	0.007	
	CXCL2	0.061	1.063 (1.025–1.102)	0.001	
	IL-8	0.015	1.016 (1.004–1.027)	0.008	

OR, odds ratio; CI, confidence interval; TBIL, total bilirubin; NLR, neutrophil-to-lymphocyte ratio.

In addition, the multivariate analysis of the serum cytokines indicated that CXCL2 (OR = 1.024, 95% CI = 1.009–1.039, *p* = 0.001), IL-6 (OR = 1.023, 95% CI = 1.000–1.047, *p* = 0.054), and IL-8 (OR = 1.014, 95% CI = 1.006–1.022, *p* < 0.001) were independent predictors of 90-day mortality. These factors were therefore selected to establish an immune prognostic model using the following formula: Ditan immune model = −5.379 + 0.024 × CXCL2 + 0.023 × IL-6 + 0.014 × IL-8.

To develop a composite immune-clinical prognostic model, the independent predictors (i.e., age, TBIL, NLR, CXCL2, IL-6, and IL-8) were subjected to multivariate logistic analysis. As shown in **Table 3**, age (OR = 1.149, 95% CI = 1.036–1.274, *p* = 0.008), TBIL (OR = 1.014, 95% CI = 1.004–1.023, *p* = 0.007), CXCL2 (OR = 1.063, 95% CI = 1.025–1.102, *p* = 0.001), and IL-8 (OR = 1.016, 95% CI = 1.004–1.027, *p* = 0.008) were included in the construction of the immune-clinical prognostic model using the following formula: Ditan immune-clinical model = −21.820 + 0.139 × Age + 0.013 × TBIL (μmol/L) + 0.061 × CXCL2 + 0.015 × IL-8.

### 3.5 Performance of the immune-clinical model compared with other models

**Figure 3:**
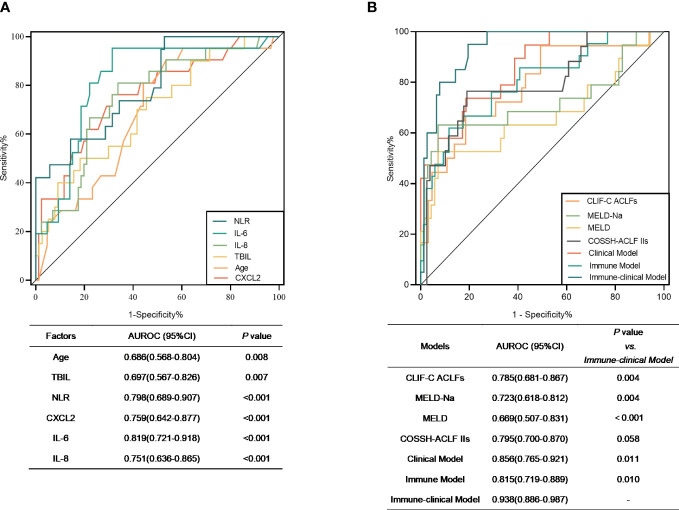
**(A)** Receiver operating characteristic (ROC) curves of the significant factors that distinguished non-survivors from survivors. **(B)** ROC curves of Model for End-Stage Liver Disease (MELD), MELD-Na, and Chronic Liver Failure Consortium on Acute-on-Chronic Liver Failure (CLIF-ACLFs) and the three models in the prognosis prediction of hepatitis B virus-related acute-on-chronic liver failure (HBV-ACLF). Clinical model = −8.967 + 0.088 × Age + 0.005 × TBIL (μmol/L) + 0.457 × NLR. Immune model = −5.379 + 0.024 × CXCL2 + 0.023 × IL-6 + 0.014 × IL-8. Immune-clinical model = −21.820 + 0.139 × Age + 0.013 × TBIL (μmol/L) + 0.061 × CXCL2 + 0.015 × IL-8. *AUROC*, area under the receiver operating characteristic curve; *TBIL*, total bilirubin; *NLR*, neutrophil-to-lymphocyte ratio.

The predictive ability of the different predictors and models to determine the mortality risk of patients with HBV-ACLF over 90 days is presented in **Figure 3**. The AUROCs of age, TBIL, NLR, CXCL2, IL-6, and IL-8 were 0.686, 0.697, 0.798, 0.759, 0.819, and 0.751, respectively (**Figure 3A**). The AUROC of the Ditan immune-clinical model was 0.938 (95% CI = 0.886–0.987), which was greater than those of the models constructed for the Ditan clinical model (AUROC = 0.856, 95% CI = 0.765–0.921), the Ditan immune model (AUROC = 0.815, 95% CI = 0.719–0.889), MELD (AUROC = 0.699, 95% CI = 0.507–0.831), MELD-Na (AUROC = 0.723, 95% CI = 0.618–0.812), and CLIF-C ACLF (AUROC = 0.785, 95% CI = 0.681–0.867; *p* < 0.05), as well as that of COSSH-ACLF II (AUROC = 0.795, 95% CI = 0.700–0.870; *p* = 0.058) (**Figure 3B**).

When the best cutoff value of −2.0 was used for the immune-clinical model, the sensitivity was 95.00% and the specificity was 80.52%, which were significantly higher than those of the other models (*p* < 0.05) (**Supplementary Table S3**). The mortality risk of patients with a Ditan immune-clinical score above the cutoff value was significantly poorer at 28 days (28.6% vs. 1.4%) and at 90 days (57.2% vs. 1.4%) compared to that of patients with a lower score (both *p* < 0.0001).

## 4 Discussion

Cytokines include a suite of peptides secreted by neutrophils, lymphocytes, and macrophages. They are involved in a wide range of biological activities, including the promotion of target cell proliferation and differentiation and the stimulation of inflammatory processes, among others, which play an important role in numerous diseases (17). Systemic inflammation is prominent in patients with ACLF and is associated with its severity and mortality (18, 19). The pathogenesis of ACLF involves the activation of the innate immune system, the release of a large number of cytokines, subsequent excessive compensatory anti-inflammatory response, the depletion of immune substances, and metabolic dysfunction. However, the pathogenesis of ACLF has not been fully elucidated. In addition, most of the current therapies for HBV-ACLF, other than liver transplantation, consist mainly of antiviral therapy and supportive measures that do not target the potential pathogenesis. Characterization of the immune profile could provide novel insights into the role of cytokines and chemokines in the pathogenesis of ACLF. However, to date, several studies on ACLF have examined only one or two cytokines or chemokines, while only a few have studied the immune and inflammatory profiles.

In this study, the use of the multiplex bead array was reported for the first-time in patients with HBV-ACLF. Both the PCA and PLS-DA methods demonstrated a clear difference in the serum cytokine/chemokine profiles between patients with different outcomes. Notably, 40 immunological cytokines were detected using the Luminex technique, and 14 cytokines were found to be associated with prognosis, with the multivariate analysis showing that IL-6, IL-8, and CXCL2 were independent immune predictors of 90-day mortality. As has been reported, inflammatory cytokines, e.g., IL-6 and IL-8, are known as important inducers of the occurrence and development of acute inflammation (20). Elevated levels of serum IL-6 and IL-8 indicated the higher risk of occurrence and mortality of HBV-ACLF (21–23). In addition, in a variety of clinical studies and experimental models, there is mounting evidence showing that neutrophils significantly contribute to tissue damage and that chemokines could activate the chemotaxis and motility of neutrophils (24, 25). In the present study, higher levels of CXCL2 and IL-8 (also known as CXCL8), the chemotactic factors for neutrophils, were observed in HBV-ACLF patients with poor prognosis, which was consistent with some previous reports (24).

Furthermore, a composite immune-clinical prognostic model, which included two immune indices (IL-8 and CXCL2) and two clinical indices (age and TBIL), was developed according to the results of the multivariate logistic regression analysis, which showed the highest predictive accuracy (0.938) compared to the MELD, MELD-Na, COSSH-ACLF II, and CLIF-C ACLF scores. The mortality risk of patients with a score −2.0 or greater was significantly higher than that of patients with a lower score at 28 days (28.6% vs. 1.4%) and at 90 days (57.2% vs. 1.4%).

This study has some limitations. Firstly, it was conducted in a single center with a limited sample size, and it is necessary to validate our findings in multiple centers. Secondly, we only collected samples from patients once for testing, and it is necessary to monitor the patients’ serum cytokines/chemokines dynamically. Finally, the study did not include healthy individuals, and it is necessary to compare the cytokine/chemokine profiles of healthy individuals with those of patients with HBV-ACLF.

In summary, this report demonstrates that the serum cytokine/chemokine profiles could distinguish HBV-ACLF patients with different prognoses. As markers for prognosis, IL-8 and CXCL2 are the key inflammatory mediators, while age and TBIL are the key clinical indices. The Ditan immune-clinical model we proposed results in accurate prognostic estimates, which could be useful as a tool for identifying patients at high risk of death and in guiding monitoring and treatment decisions.

## Data availability statement

The original contributions presented in the study are included in the article/**Supplementary Material**. Further inquiries can be directed to the corresponding author.

## Ethics statement

The studies involving human participants were reviewed and approved by the Ethics Committee of the Beijing Ditan Hospital. The patients/participants provided written informed consent to participate in this study.

## Author contributions

XW and FG contributed to the study conception and design. BZ, FG, YL, KS, and YH performed material preparation, data collection, and analysis. QZ and JC provided support for the analysis of data. BZ wrote the first draft of the manuscript. All authors commented on previous versions of the manuscript. All authors contributed to the article and approved the submitted version.
