# Supplementary material for: Serum cytokine and chemokine profiles and disease prognosis in hepatitis B virus-related acute-on-chronic liver failure
Source: Front Immunol. 2023 Apr 27;14:1133656. doi: 10.3389/fimmu.2023.1133656 (PMC10172591; doi:10.3389/fimmu.2023.1133656)
Supplement: Supplementary file 1 [file Table_1.docx]

**Supplementary table 1** Logistic multivariate regression analysis of 9 clinical factors.

|  | β | OR (95% CI) | *P* |
| --- | --- | --- | --- |
| Age | 0.088 | 1.092 (1.021-1.167) | 0.034^#^ |
| HE (%) | 0.167 | 1.182 (0.460-3.036) | 0.728 |
| SBP (%) | 0.147 | 1.158 (0.241-5.562) | 0.855 |
| TBIL, μmol/L | 0.005 | 1.005 (1.000-1.010) | 0.045^#^ |
| Na, mmol/L | -0.015 | 0.985 (0.797-1.217) | 0.888 |
| Cr, μmol/L | 0.013 | 1.013 (0.991-1.035) | 0.251 |
| NLR | 0.457 | 1.579 (1.163-2.145) | 0.003^#^ |
| MLR | 0.109 | 1.115(0.849-1.464) | 0.434 |
| PTA, % | 0.005 | 1.005(0.935-1.081) | 0.891 |
| *Constant* | -8.967 | 0.000 | ＜0.001^#^ |

OR, odds ratio. CI, confidence interval. * Univariate analysis was screened at *P*<0.05；# multifactor regression analysis was performed with the backward step likelihood ratio method, *P*<0.05 was statistically significant. To avoid the co-linearity of several factors (WBC and NC and NLR; MC and MLR; PT and PTA and INR), we included age, HE, SBP, TBIL, Na, Cr, NLR, MLR, PTA for multifactor regression analysis.
