# Supplementary material for: Serum cytokine and chemokine profiles and disease prognosis in hepatitis B virus-related acute-on-chronic liver failure
Source: Front Immunol. 2023 Apr 27;14:1133656. doi: 10.3389/fimmu.2023.1133656 (PMC10172591; doi:10.3389/fimmu.2023.1133656)
Supplement: Supplementary file 2 [file Table_2.docx]

**Supplementary table 2** Logistic multivariate regression analysis of 14 cytokines and chemokines.

|  | β | OR (95% CI) | *P* |
| --- | --- | --- | --- |
| IL-1β | 0.670 | 1.129(0.503-2.034) | 0.189 |
| IL-6 | 0.023 | 1.023(1.000-1.047) | 0.054 |
| IL-8 | 0.014 | 1.014(1.006-1.022) | ＜0.001^#^ |
| IL-10 | 0.026 | 1.026(0.928-1.134) | 0.616 |
| IFN-γ | 0.028 | 1.028(0.971-1.089) | 0.345 |
| TNF-α | 0.008 | 1.008(0.977-1.039) | 0.538 |
| GMCSF | 0.007 | 1.007(0.915-1.107) | 0.891 |
| CCL21 | 0.001 | 1.001(0.996-1.006) | 0.817 |
| CCL23 | 0.002 | 1.002(0.997-1.006) | 0.447 |
| CX3CL1 | -0.005 | 0.995(0.986-1.004) | 0.258 |
| CXCL1 | 0.011 | 1.011(0.984-1.039) | 0.416 |
| CXCL2 | 0.024 | 1.024(1.009-1.039) | 0.001^#^ |
| CXCL9 | 0.001 | 1.0011(0.997-1.005) | 0.602 |
| CXCL13 | 0.023 | 1.023(0.994-1.052) | 0.116 |
| *Constant* | -5.379 | 0.005 | ＜0.001^#^ |

OR, odds ratio. CI, confidence interval. * Univariate analysis was screened at *P*<0.05；# multifactor regression analysis was performed with the backward step likelihood ratio method, *P*<0.05 was statistically significant.
