# Supplementary material for: Serum cytokine and chemokine profiles and disease prognosis in hepatitis B virus-related acute-on-chronic liver failure
Source: Front Immunol. 2023 Apr 27;14:1133656. doi: 10.3389/fimmu.2023.1133656 (PMC10172591; doi:10.3389/fimmu.2023.1133656)
Supplement: Supplementary file 3 [file Table_3.docx]

**Supplementary table 3** Predictive performance indices and the comparison of 6 prediction scores.

| Score | AUROC (95%CI) | *P* value | Cutoff | Sensitivity% | Specificity% | Youden index | +LR | -LR | Statistic | *P*^a)^ |
| --- | --- | --- | --- | --- | --- | --- | --- | --- | --- | --- |
| CLIF-C ACLF | 0.785(0.681-0.867) | ＜0.001 | 39.50 | 66.67 | 81.54 | 48.21 | 3.61 | 0.41 | Z=2.890 | 0.004 |
| MELD-Na | 0.723(0.618-0.812) | 0.003 | 27.11 | 63.16 | 92.86 | 56.02 | 8.84 | 0.40 | Z=2.895 | 0.004 |
| MELD | 0.669(0.507-0.831) | 0.024 | 26.61 | 52.63 | 92.86 | 45.49 | 7.37 | 0.51 | Z=3.554 | <0.001 |
| COSSH-ACLF Ⅱs | 0.795(0.700-0.870) | ＜0.001 | 6.79 | 76.47 | 81.01 | 57.48 | 4.03 | 0.29 | Z=4.326 | 0.058 |
| Clinical Model | 0.856(0.765-0.921) | ＜0.001 | 54.12 | 73.68 | 81.43 | 55.11 | 3.97 | 0.32 | Z=2.547 | 0.011 |
| Immune Model | 0.815(0.719-0.889) | ＜0.001 | 30.22 | 66.67 | 83.72 | 50.39 | 4.10 | 0.40 | Z=2.590 | 0.010 |
| Immune-clinical Model | 0.938(0.886-0.987) | ＜0.001 | -2.09 | 95.00 | 80.52 | 75.52 | 4.88 | 0.06 | - | - |

Clinical Model = -8.967 + 0.088 × Age + 0.005 × TBIL (μmol/L) + 0.457 × NLR.

Immune Model = -5.379 + 0.024 × CXCL2 + 0.023 × IL-6 + 0.014 × IL-8.

Immune-clinical Model = -21.820 + 0.139 × Age + 0.013×TBIL (μmol/L) + 0.061×CXCL2 + 0.015 × IL-8.

a) *P* value of AUROC comparison between different models and P3. +LR, positive likelihood ratio; -LR, negative likelihood ratio. AUROC, area under the receiver operating characteristic curve.
